# Distribution and molecular characterization of ESBL, pAmpC β-lactamases, and non-β-lactam encoding genes in Enterobacteriaceae isolated from hospital wastewater in Eastern Cape Province, South Africa

**DOI:** 10.1371/journal.pone.0254753

**Published:** 2021-07-21

**Authors:** Folake Temitope Fadare, Anthony Ifeanyi Okoh

**Affiliations:** 1 SAMRC Microbial Water Quality Monitoring Centre, University of Fort Hare, Alice, South Africa; 2 Department of Biochemistry and Microbiology, Applied and Environmental Microbiology Research Group, University of Fort Hare, Alice, South Africa; University of Nicolaus Copernicus in Torun, POLAND

## Abstract

Globally, there is an increasing occurrence of multidrug-resistant (MDR) Enterobacteriaceae with extended-spectrum β-lactamases (ESBLs) and/or plasmid-encoded AmpC (pAmpC) β-lactamases in clinical and environmental settings of significant concern. Therefore, we aimed to evaluate the occurrence of ESBL/pAmpC genetic determinants, and some essential non-β-lactam genetic determinants in the MDR phenotypic antimicrobial resistance in Enterobacteriaceae isolates recovered from hospital wastewater. We collected samples from two hospitals in Amathole and Chris Hani District Municipalities in the Eastern Cape Province, South Africa, within October and November 2017. Using the matrix-assisted laser desorption ionization-time of flight mass spectrometry (MALDI-TOF), we identified a total of 44 presumptive Enterobacteriaceae isolates. From this, 31 were identified as the targeted Enterobacteriaceae members. Thirty-six percent of these belonged to *Klebsiella oxytoca*, while 29% were *Klebsiella pneumoniae*. The other identified isolates included *Citrobacter freundii* and *Escherichia coli* (10%), *Enterobacter asburiae* (6%), *Enterobacter amnigenus*, *Enterobacter hormaechei*, and *Enterobacter kobei* (3%). We established the antibiotic susceptibility profiles of these identified bacterial isolates against a panel of 18 selected antibiotics belonging to 11 classes were established following established guidelines by the Clinical and Laboratory Standard Institute. All the bacterial species exhibited resistance phenotypically against at least four antibiotic classes and were classified as MDR. Notably, all the bacterial species displayed resistance against cefotaxime, ampicillin, nalidixic acid, and trimethoprim-sulfamethoxazole. The generated multiple antibiotic resistance indices ranged between 0.5 to 1.0, with the highest value seen in one *K*. *oxytoca* isolated. Molecular characterization via the Polymerase Chain Reaction uncovered various ESBLs, pAmpCs, and other non-β-lactam encoding genes. Of the phenotypically resistant isolates screened for each class of antibiotics, the ESBLs detected were *bla*_CTX-M_ group (including groups 1, 2, and 9) [51.6% (16/31)], *bla*_TEM_ [32.3% (10/31)], *bla*_OXA-1-like_ [19.4% (6/31)], *bla*_SHV_ [12.9% (4/31)], *bla*_PER_ [6.5% (2/31)], *bla*_VEB_ [3.2% (1/31)], *bla*_OXA-48-like_ and *bla*_VIM_ [15.4% (2/13)], and *bla*_IMP_ [7.7% (1/13)]. The pAmpC resistance determinants detected were *bla*_CIT_ [12.9% (4/31)], *bla*_FOX_ [9.7% (3/31)], *bla*_EBC_ [6.5% (2/31)], and *bla*_DHA_ [3.2% (1/31)]. The frequencies of the non-β-lactam genes detected were *catII* [79.2% (19/24)], *tetA* [46.7% (14/30)], *sulI* and *sulII* [35.5% (11/31)], *tetB* [23.3% (7/30)], *aadA* [12.9% (4/31)], *tetC* [10% (3/30)], and *tetD* [3.3% (1/30)]. These results indicate that hospital wastewater is laden with potentially pathogenic MDR Enterobacteriaceae with various antibiotic resistance genes that can be spread to humans throughout the food chain, provided the wastewaters are not properly treated before eventual discharge into the environment.

## Introduction

The hospital setting and its resultant waste can be regarded as significant hot spots for the presence and potential distribution of antibiotic-resistant bacteria (ARB) [1,2]. Apart from the high bacterial loads commonly reported in hospital effluents, they also contain sub-therapeutic antibiotic concentrations [3]. Hospital wastewater can serve as a reservoir of ARB and antibiotic resistance genes (ARGs) due to massive quantities of antibiotics used in hospitals for prevention and treatment purposes. Unfortunately, the body does not absorb most of these antibiotics entirely and are often expelled as waste into the environment [4]. This scenario can foster the development and subsequent propagation of ARGs among bacterial species, thereby compromising public health safety due to incorrect waste disposal [5]. In many developing nations, such as South Africa, there are currently no defined regulations regarding a pre-treatment process for hospital wastes before their release into the municipal wastewater treatment plants (WWTPs). This situation exerts pressure on the existing WWTPs, designed ab initio to reduce and remove the bacterial load. The WWTPs, even at optimal functioning capacity, have been reported to expedite the movement of ARGs among bacterial species and have been subsequently highlighted as potential public health risks [3,6]. The World Health Organization (WHO) [7] has forecast that antibiotic-resistant disease-causing microorganisms will result in an annual death of 10 million people globally by 2050 if concerted efforts are not put in place to forestall this impending global antimicrobial resistance (AMR) scourge. This forecast is a significant concern in South Africa, where a substantial fraction of the country’s population has weakened immune systems. Other concerning issues include poor hygiene and inadequate infrastructure which could make the people more vulnerable to infectious illnesses, thereby contributing to a higher risk of death due to AMR and damage to the economy [8].

The occurrences of these bacterial species, especially the antibiotic-resistant phenotypes in the environment and hospital, are an essential environmental and public health concern [9,10]. The development of multidrug-resistant (MDR) members of the Enterobacteriaceae family, most notably the *Klebsiella pneumoniae* and *K*. *oxytoca*, is quite concerning. These Klebsiella species are widely known as opportunistic hospital pathogens, which can escape from the hospital environment via wastes [1,11]. Other clinically significant genera of this family include food-related pathogens such as *Salmonella* spp. and *Escherichia coli* and opportunistic pathogenic organisms such as *Citrobacter* spp. and *Enterobacter* spp. These bacterial species are vital causal agents of infections such as diarrhea, pneumonia, blood and wound infections, urinary tract infections, and bowel inflammations [12–14].

There are various mechanisms in which bacterial species exhibit AMR to antimicrobials administered against them. Among the Enterobacteriaceae, the manufacturing of β-lactamases is considered an essential means of AMR. These enzymes can break the β-lactam ring, the chemical backbone in the β-lactam antibiotic class. These include penicillins, carbapenems, and cephalosporins prescribed to treat infections arising from Enterobacteriaceae. Enterobacteriaceae’ ability to produce the enzymes ESBLs and the pAmpC β-lactamases are a cause of worry to public health safety [10,12,15,16]. According to Bush and Jacoby [17], the ESBLs are the Ambler class A, including the TEM-, SHV-, OXA- and CTX-M enzymes. At least 200 SHV and TEM variants have been previously reported, while 90 different enzymes in the CTX-M types have also been documented. In the 1980s, the most common ESBLs were the TEM, while since the turn of the 21^st^ century, the CTX-M types became more predominant. Other ESBLs described are the PER, VEB, and GES [18]. The AmpC β-lactamases are the class C enzymes, and they differ from the ESBLs. Unlike the class A enzymes, AmpC β-lactamases are active against an additional β-lactam antibiotic class cephamycin (cefoxitin). They cannot be made inactive by clavulanic acid, the class A enzyme inhibitor [17,19]. The pAmpC-producers are different from the chromosomal AmpC because they are not inducible [20]. The pAmpC belongs to six families, including DHA, FOX, ACC, CIT, EBC, and MOX, with the first two families being the most commonly detected [21].

The resistance of ESBL producers against multiple antibiotic classes makes them of significant concern in the clinical settings [18,22], making the treatment of patients infected by them difficult and quite often almost impossible. Additionally, the co-occurrence of β-lactamases, particularly the pAmpC β-lactamases, and the ESBLs has been commonly reported [10,21]. The genes encoding the ESBLs and pAmpC are frequently encoded on plasmids with large sizes. These plasmids concurrently encode resistance genes to the non-β-lactams. These include other classes of antibiotics such as aminoglycosides, phenicols, tetracyclines, trimethoprim, and sulphonamides. Therefore, we are reporting the presence of ESBL/pAmpC, and some essential non-β-lactam genetic determinants in the MDR phenotypic AMR profiles of Enterobacteriaceae isolates retrieved from hospital wastewater in two District Municipalities in South Africa.

## Materials and methods

### Study sites

We collected samples from two hospitals situated in Amathole District Municipality (ADM) and Chris Hani District Municipality (CHDM) in the Eastern Cape Province (ECP), Republic of South Africa (RSA). The population size is estimated at 840,055 and 880,790 in CHDM and ADM, respectively. The two hospitals are deemed rural district hospitals with an approximately 150-bed capacity.

### Sample collection and processing of target bacterial species

Grab water samples were collected in triplicates from the hospital in CHDM in October, while samples from ADM was retrieved in November 2017. Samples were collected by lowering a sterile 2 L glass bottle attached to a string of appropriate length lowered to a maintenance hole where the hospital wastewater contains before being discharged into the main municipal wastewater stream. Samples were preserved on ice until processing within hours of collection. Each sample was processed in triplicates. A ten-fold serial dilution was carried out using sterile distilled water. One hundred mL of the adequately diluted samples were filtered under a vacuum. The membrane filters were transferred unto Violet Red Bile Glucose (VRBG) agar (Merck, Modderfontein, RSA). For the isolation of presumptive Enterobacteriaceae, the VRBG plates were incubated at 37 °C for 18 hours. For the isolation of presumptive *Citrobacter* spp., *E*. *coli*, *Enterobacter* spp., and *Klebsiella* spp., the different colonies on VRBG were then picked unto Eosin Methylene Blue agar and MacConkey agar (Conda, Pronadisa, RSA). A total of 44 presumptive isolates were stored at -80 °C in 25% glycerol stock for further laboratory analysis.

### Characterizing bacterial isolates

Purified presumptive isolates were identified with the aid of Matrix-Assisted Laser Desorption Ionization Time of Flight coupled with Mass Spectrometry (MALDI-TOF MS) Biotyper 3.0 protocol (Bruker Daltonics, Germany) to species level as previously described [23]. All isolates were tested in duplicates. For quality control, bacterial Test Standard (BTS) (8255343) was included in every plate. The obtained results were interpreted following the manufacturer’s guidelines. Score values lower than 1.70 were not included, as the identification is deemed unreliable [23].

### Antimicrobial susceptibility disk diffusion assay

Upon MALDI-TOF identification, 31 of the isolates which belonged to the targeted bacterial species recovered from the sampling sites were subjected to antimicrobial susceptibility testing. The disk diffusion protocol, according to the established guidelines by the Clinical and Laboratory Standards Institute guidelines (CLSI) [24], was employed. In brief, the identified bacterial species were selected from 18 hours old pure cultures and dispersed into normal saline. The resulting solution was then adjusted where necessary to 0.5 McFarland turbidity standards. The standardized test solution was evenly distributed on the Mueller-Hinton agar (Merck, Johannesburg) with a sterilized swab. The relevant antibiotic disks were implanted using the disc dispensing apparatus. After fifteen minutes, the inoculated plates were inverted and incubated at 37 °C for 18 hours. The inhibition zones were measured in millimeters, and results were interpreted as "Resistant (R), Intermediate (I), or Susceptible (S)" using the CLSI cutoff point. In contrast, the zone diameter of *E*. *coli* ATCC 25922 was used to interpret the results obtained for the antibiotic class Polymyxins. We used a panel of 18 antibiotics that are classified into eleven antibiotics classes. These antibiotics are often recommended through the Center for Disease Control and Prevention (CDC) to treat infections mediated by Enterobacteriaceae. The antibiotics were; doxycycline (DXT:30 μg) and tetracycline (T:30 μg) belonging to the tetracyclines, ampicillin (AP:10 μg) and amoxicillin/clavulanic acid (AUG:30 μg) belonging to the β-lactams, amikacin (AK:30 μg) and gentamicin (GM:10 μg) belonging to aminoglycosides, nitrofurantoin (NI:300 μg) belonging to the nitrofurans, cefuroxime (CXM:30 μg) and cefotaxime (CTX:30 μg) belonging to the cephems, norfloxacin (NOR:30 μg) and ciprofloxacin (CIP:5 μg) belonging to the fluoroquinolones, chloramphenicol (C:30 μg) belonging to the phenicols, nalidixic acid (NA:30 μg) belonging to the quinolones, colistin sulphate (CO:25 μg) and polymyxin B (PB:300 units) belonging to the polymyxins, trimethoprim-sulfamethoxazole (TS:25 μg) belonging to the sulphonamides, and lastly, imipenem (IMI:10 μg) and meropenem (MEM:10 μg) belonging to the carbapenems (Mast Diagnostics, UK).

### Assessment of multiple antibiotic resistance phenotype (MARP) and multiple antibiotic resistance index (MARI)

Bacterial species investigated were regarded as MDR when they exhibited resistance against a minimum of 3 different classes of antibiotics. The MARPs for all the bacterial species were assessed because they exhibited resistance against three or more than antibiotic classes out of the eleven classes as earlier described [25]. The MARI of the bacterial species was derived as described by [26] following the mathematical equation:

MARindex=b/c,

Here ’’b’’ indicates the sum of antibiotics against which the bacterial species displayed resistance, while ’’c’’ indicates the total of antibiotics used against the bacterial species. When the MARI is higher in value than 0.2, it indicates that antibiotics are being used intensively in that area and implies an environment with a high-risk of AMR’s proliferation [26,27].

### DNA extraction

Isolates preserved in glycerol stocks were resuscitated in nutrient broth (Merck, South Africa) and incubated aerobically under shaking conditions at 200 rpm at 37 °C overnight. The genomic DNA extraction was carried out via the boiling method, as previously stated [28], with a bit of modification. A volume of 100 μl of DNase/RNase-free water was dispensed into sterile Eppendorf tubes, into which pure overnight colonies were dispersed. The resulting suspension was vortexed and boiled at 100 °C for 10 min. The solution was immediately placed on ice, and the cell lysate was extracted after centrifugation at 13,000 g for 4 min via the PRISMR micro-centrifuge (Labnet International, USA). This supernatant contained the DNA utilized in polymerase chain reaction (PCR) assays.

### Antibiotic resistance genes screening

All confirmed targeted Enterobacteriaceae’ members were investigated for the existence of a variety of relevant ARGs via the PCR technique. Thirty-one ARGs investigated were classified into three different groups. These are the ESBL resistance determinants (13); *bla*_CTX-M_ (inclusive of groups 1, 2, and 9), *bla*_TEM_, *bla*_SHV_, *bla*_GES_, *bla*_IMP_, *bla*_KPC_, *bla*_VIM_, *bla*_OXA-1-like_, *bla*_PER_, *bla*_OXA-48-like_, and *bla*_VEB_ the pAmpC resistance genes (6); *bla*_ACC_, *bla*_EBC_, *bla*_FOX_, *bla*_CIT_, *bla*_DHA_, and *bla*_MOX_, and non-β-lactam resistance determinants (12); *aadA*, *catI*, *catII*, *strA*, *sulI*, *sulII*, *tetA*, *tetB*, *tetC*, *tetD*, *tetK*, and *tetM*. Any isolate that displayed a full or an intermediate resistance phenotype was investigated for the corresponding ARGs. Conventional singleplex, duplex and multiplex PCR was performed employing a Biorad Thermal Cycler (USA). All primers were manufactured by Inqaba Biotechnological Industries (Pretoria, South Africa). The specific target genes, their primers, thermocycling conditions, and the expected molecular weight are described in S1 Table. Each reaction tube consisted of 12.5 μl PCR master mix (Thermo Scientific, USA), 1 μl of each primer pair, 5.5 μl of DNase/RNase-free water, and 5 μl genomic DNA. Negative controls were constituted as other reactions, but the genomic DNA was substituted with sterilized buffer. The 5 μl of the amplified DNA were resolved in a 1.5% (w/v) agarose gel (Separations, South Africa) stained with 5 μl ethidium bromide (0.001 μg/ml). A molecular marker of 100 base pairs (Thermo Scientific, USA) was used. The gel electrophoresis was run in 0.5X Tris-borate EDTA buffer at 100 volts for 1 hour and visualized by a UV transillumination (ALLIANCE 4.7, UVIec, Merton, London, UK).

### Data analysis

The data obtained were analyzed using the descriptive statistical package available in Microsoft Excel 2010 (USA).

## Results

### MALDI-TOF identification and characterization of bacterial species

**Fig 1:**
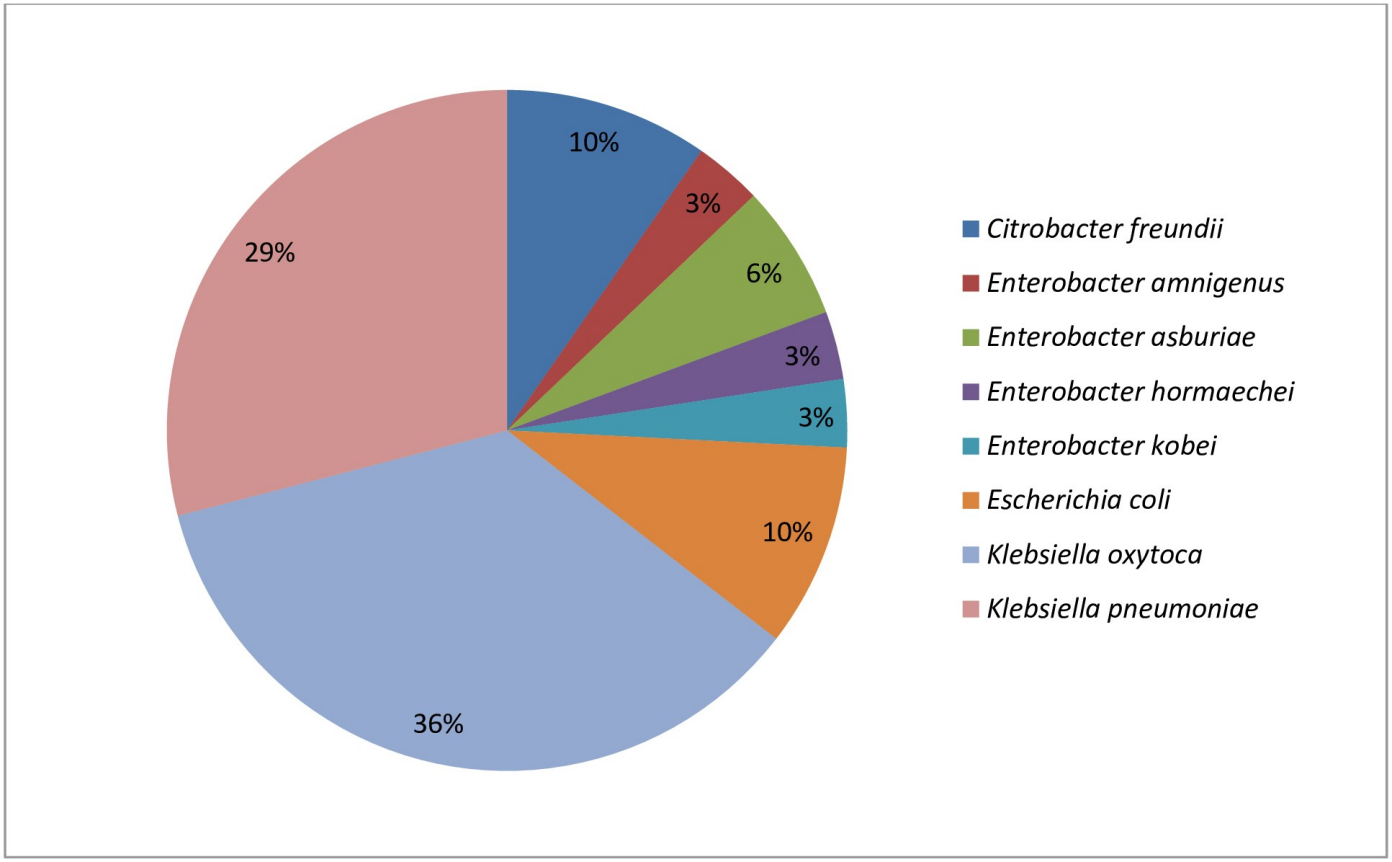
The distribution of the targeted Enterobacteriaceae’ members (n = 31) retrieved from hospital wastewaters.

Presumptive isolates (n = 44) from the different selective agar used belonged to 2 families, namely Moraxellaceae (4) and Enterobacteriaceae (35). The remaining five bacterial species had a matching value lower than 1.7 and were excluded from this study as their identities were not reliable. Of the Enterobacteriaceae family, six genera were identified, which include Citrobacter (3), Enterobacter (5), Escherichia (3), Klebsiella (20), Serratia (3), and Raoultella (1). The identities of the presumptive isolates are as shown in S2 Table. This study, however, focuses on the first four genera of the Enterobacteriaceae family, totalling 31. The most prevalent bacterial species among these was *K*. *oxytoca*, with 36%, followed by *K*. *pneumoniae* with 29%. The *Enterobacter* spp. had four species, which include *E*. *amnigenus*, *E*. *asburiae*, *E*. *hormaechei*, and *E*. *kobei*. Among the genera Citrobacter and Escherichia, the species identified were *C*. *freundii* and *E*. *coli*, respectively. Fig 1 shows the identity and the distribution of the targeted bacteria isolates.

### Antimicrobial susceptibility profiles

**Fig 2:**
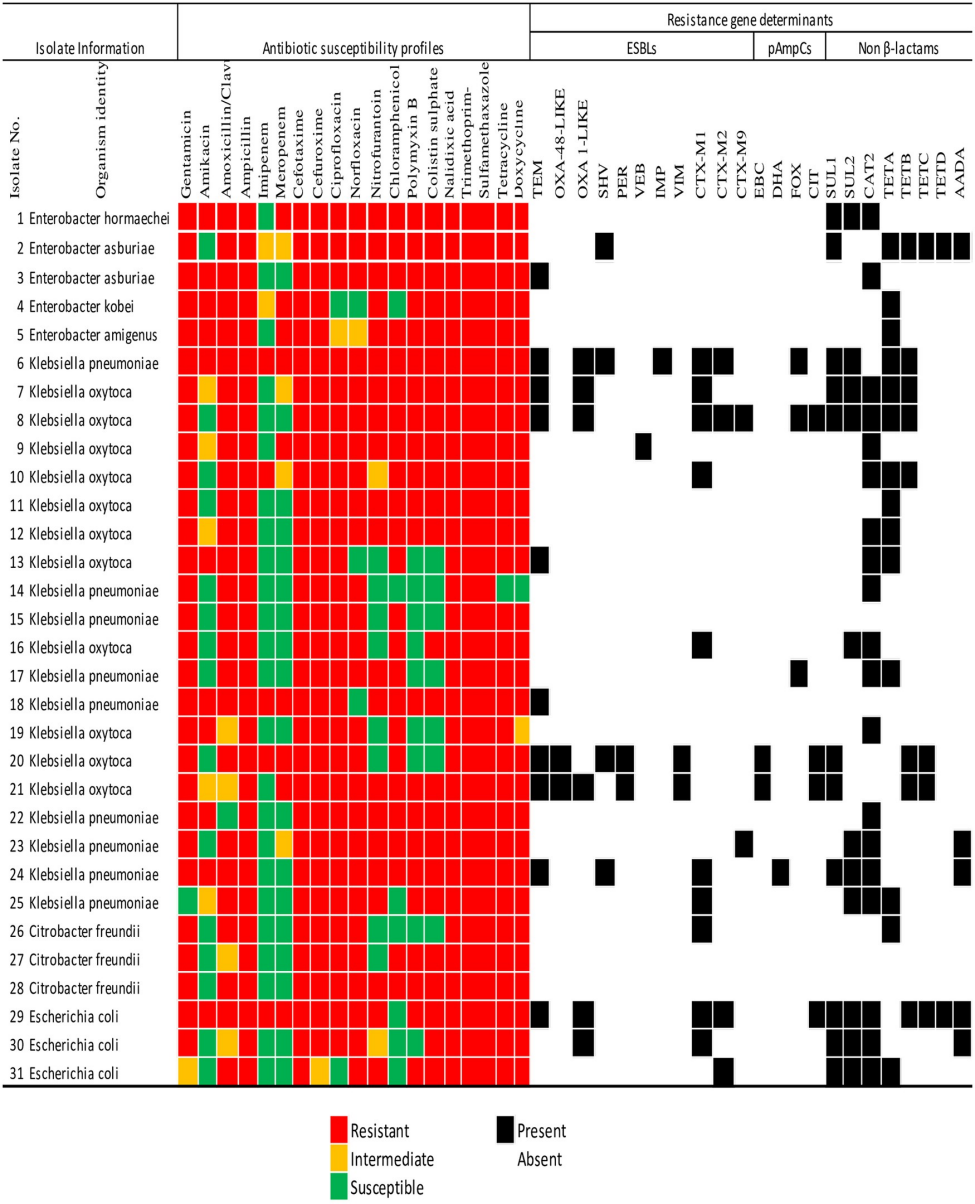
Antimicrobial susceptibility profile of Enterobacteriaceae recovered from hospital wastewater indicating the Extended-Spectrum-β-Lactamase, plasmid-encoded AmpC β-lactamase and other non-β-lactam genetic resistance determinants. The colour code of the antimicrobial susceptibility profiles indicates the phenotypes of the isolates to specific antibiotics in 11 different classes. The detection of the resistance gene determinants is shown as present or absent.

Multidrug resistance was seen in all the bacterial species investigated, as shown in Fig 2. Notably, all the isolates displayed full resistance against a minimum of four antibiotics belonging to four antibiotic classes. These include trimethoprim-sulfamethoxazole, ampicillin, cefotaxime, and nalidixic acid belonging to antibiotic classes sulphonamides, β-lactams, cephems, and quinolones, respectively. The next dominant resistance was observed against cefuroxime, tetracycline, and doxycycline, with 96.8%. We noted a high rate of non-susceptibility against all the tested antibiotics. The least resistance was observed against the class carbapenems, with 16.1% and 29% of the bacterial species exhibiting resistance against imipenem and meropenem. The resistance frequencies of other antibiotics were gentamicin (93.5%), ciprofloxacin (90.3%), norfloxacin (87.1%), amoxicillin/clavulanic acid (83.9%), chloramphenicol and colistin sulfate (77.4%), polymyxin B (71%), nitrofurantoin (67.7%), and amikacin (35.5%).

Diverse antimicrobial susceptibility profiles were observed among all the bacterial species. As shown (Fig 2), most bacterial strains displayed varying phenotype to each of the antibiotics tested. This scenario might be due to the variable genetic characteristic inherent in each bacterial strain. It is essential to note that a *K*. *oxytoca* (isolate No. 6) exhibited resistance against all the tested eighteen antibiotics, carbapenems inclusive.

All the five *Enterobacter* spp. from this niche were resistant against 12 of the 18 antibiotics used in this study. These antibiotics are gentamicin, amoxicillin/clavulanic acid, ampicillin, cefotaxime, cefuroxime, nitrofurantoin, polymyxin B, colistin sulfate, nalidixic acid, trimethoprim-sulfamethoxazole, tetracycline, and doxycycline. Following was resistance against amikacin and chloramphenicol displayed by 80% of the isolates. The only antibiotic against which no resistance was observed in this genus was imipenem, a carbapenem. The *Klebsiella* spp. all exhibited resistance against six of the antibiotics used, including ampicillin, cefotaxime, cefuroxime, ciprofloxacin, nalidixic acid, and trimethoprim-sulfamethoxazole. About 95% of this genus were resistant against gentamicin and tetracycline, while 20% of the isolates were resistant against imipenem. The *E*. *coli* isolates showed resistance against eight tested antibiotics, including ampicillin, cefotaxime, norfloxacin, colistin sulfate, nalidixic acid, trimethoprim-sulfamethoxazole, tetracycline, and doxycycline. However, there was no resistance observed against chloramphenicol. For *Citrobacter* spp., all the isolates exhibited resistance against ten out of the antibiotics tested, including gentamicin, ampicillin, cefotaxime, cefuroxime, ciprofloxacin, norfloxacin, nalidixic acid, trimethoprim-sulfamethoxazole, tetracycline, and doxycycline. At the same time, there was no resistance observed against antibiotics amikacin, imipenem, and meropenem.

### Multiple antibiotic resistance phenotypes (MARPs) and multiple antibiotic resistance index (MARI) of targeted Enterobacteriaceae from hospital wastewater

The different MARPs and the MARIs displayed by *E*. *coli*, *Citrobacter* spp., *Enterobacter* spp. and recovered from hospital wastewater samples are presented in Table 1, while *Klebsiella* spp. is presented in Table 2. All the bacterial species exhibited a phenotypic resistance against a minimum of nine out of the 18 test antibiotics. We observed a maximum resistance in a *K*. *oxytoca*, which displayed resistance against all the antibiotics assayed. The MARI obtained from the hospital wastewaters ranged from 0.5 to 1.0 amongst all. All the phenotypes observed occurred uniquely except for a pattern among the *Klebsiella* spp. which occurred five times.

**Table 1:** The multiple antibiotic resistance phenotypes and multiple antibiotic resistance index in *Enterobacter* spp., *E*. *coli*, and *Citrobacter freundii* recovered from hospital wastewater.

*Enterobacter* species (n = 5)			
MAR phenotypes	No of antibiotics showing resistance	No of phenotype observed	MAR index
GM, AK, AUG, AP, MEM, CTX, CXM, CIP, NOR, NI, C, PB, CO, NA, TS, T, DXT	17	1	0.94
GM, AK, AUG, AP, MEM, CTX, CXM, NI, C, PB, CO, NA, TS, T, DXT	15	1	0.81
GM, AK, AUG, AP, MEM, CTX, CXM, NI, PB, CO, NA, TS, T, DXT	14	1	0.75
GM, AK, AUG, AP, CTX, CXM, CIP, NOR, NI, C, PB, CO, NA, TS, T, DXT	16	1	0.88
GM, AUG, AP, CTX, CXM, CIP, NOR, NI, C, PB, CO, NA, TS, T, DXT	15	1	0.83
*E*. *coli* (n = 3)			
GM, AK, AUG, AP, IMI, MEM, CTX, CXM, CIP, NOR, NI, PB, CO, NA, TS, T, DXT	17	1	0.94
GM, AP, CTX, CXM, CIP, NOR, CO, NA, TS, T, DXT	11	1	0.61
AUG, AP, CTX, NOR, NI, PB, CO, NA, TS, T, DXT	11	1	0.61
*Citrobacter* species (n = 3)			
GM, AUG, AP, CTX, CXM, CIP, NOR, NI, C, PB, CO, NA, TS, T, DXT	15	1	0.83
GM,AUG, AP, CTX, CXM, CIP, NOR,NA, TS, T, DXT	11	1	0.61
GM, AP, CTX, CXM, CIP, NOR, C, PB, CO, NA, TS, T, DXT	13	1	0.72

Antibiotics code: GM–gentamicin, AK–amikacin, AM–ampicillin, AUG–amoxicillin/clavulanic acid, MEM–meropenem, IMI–imipenem, NI–nitrofurantoin, CXM–cefuroxime, CTX–cefotaxime, NOR–norfloxacin, CIP–ciprofloxacin, C–chloramphenicol, PB–polymyxin B, CO–colistin sulphate, DXT–doxycycline, T–tetracycline and TS–trimethoprim/Sulfamethoxazole.

**Table 2:** The multiple antibiotic resistance phenotypes and indices in *Klebsiella* spp. recovered from hospital wastewater.

*Klebsiella* species (n = 20)			
MAR phenotypes	No of antibiotics showing resistance	No of phenotype observed	MAR index
GM, AK, AUG, AP, IMI, MEM, CTX, CXM, CIP, NOR, NI, C, PB, CO, NA, TS, T, DXT	18	1	1.00
GM, AK, AUG, AP, IMI, MEM, CTX, CXM, CIP, NI, C, PB, CO, NA, TS, T, DXT	17	1	0.94
GM, AK, AUG, AP, CTX, CXM, CIP, NOR, NI, C, PB, CO, NA, TS, T, DXT	16	1	0.89
GM, AK, AUG, AP, CTX, CXM, CIP, C, NA, TS, T, DXT	12	1	0.67
GM, AK, AP, CTX, CXM, CIP, NOR, NI, C, PB, CO, NA, TS, T, DXT	15	1	0.83
GM, AK, AP, CTX, CXM, CIP, NOR, C, NA, TS, T	11	1	0.61
GM,AUG, AP, IMI, MEM, CTX, CXM, CIP, NOR, C, NA, TS, T, DXT	14	1	0.78
GM, AUG, AP, IMI, CTX, CXM, CIP, NOR, C, PB, CO, NA, TS, T, DXT	15	1	0.83
GM, AUG, AP, MEM, CTX, CXM, CIP, NOR, NI, C, PB, CO, NA, TS, T, DXT	16	1	0.89
GM, AUG, AP, CTX, CXM, CIP, NOR, NI, C, PB, CO, NA, TS, T, DXT	15	5	0.83
GM, AUG, AP, CTX, CXM, CIP, NOR, NI, C, NA, TS, T, DXT	13	1	0.72
GM, AUG, AP, CTX, CXM, CIP, NOR, C, CO, NA, TS, T, DXT	13	1	0.72
GM, AUG, AP, CTX, CXM, CIP, NOR, C, NA, TS, T, DXT	12	1	0.67
GM, AUG, AP,CTX, CXM, CIP, NOR, NA, TS	9	1	0.50
GM, AP, MEM, CTX, CXM, CIP, NOR, NI, C, PB, CO, NA, TS, T, DXT	15	1	0.83
AUG, AP, CTX, CXM, CIP, NOR, NI, PB, CO, NA, TS, T, DXT	13	1	0.72

^**b**^ represents antibiotics code in Table 1 footnotes.

### Genotypic antibiotic resistance signatures

From the 31 bacterial species investigated in this study, genes encoding β-lactamases were detected in 64.5% (20/31), while the non-β-lactam resistance determinants were detected in 87.1% (27/31). Within the β-lactamases, the ESBLs assayed for were detected in 58.1% (18/31) isolates, including *K*.*oxytoca* (n = 8), *E*. *asburiae* (n = 2), *K*. *pneumoniae* (n = 4), *E*. *coli* (n = 3), and *C*. *freundii* (n = 1). In comparison, the pAmpCs were detected in 22.6% (7/31), including *K*. *oxytoca* (n = 3), *E*. *coli* (n = 1), and *K*. *pneumoniae* (n = 3), as seen in Fig 2 and Table 3. There was a co-occurrence of the ESBL and pAmpC genetic determinants in 6 of the isolates. The most frequently detected ESBL resistance determinant for the β-lactams was the *bla*_CTX-M_ group (including groups 1, 2, and 3), which was detected in the isolates at a rate of 51.6% (16/31). Next was the *bla*_TEM_ gene detected in 32.3% (10/31) of the isolates. Other ESBL genes detected include *bla*_SHV_ [12.9% (4/31)], *bla*_PER_ [6.5% (2/31)], and *bla*_VEB_ [3.2% (1/31)]. Among the carbapenemases, the resistance gene determinants were *bla*_OXA-1-like_ [19.4% (6/31)], *bla*_OXA-48-like_ and *bla*_VIM_ [15.4% (2/13)], and *bla*_IMP_ [7.7% (1/13)]. The *bla*_CIT_ gene was the most detected amongst the pAmpCs, with 12.9% (4/31) of the isolates harbouring this resistance gene, followed by *bla*_FOX_ [7% (3/31)]. Others were *bla*_EBC_ [6.5% (2/31)] and *bla*_DHA_ [3.2% (1/31)]. The most frequently detected non-β-lactam resistance determinant was *CatII*, as it was detected in 79.2% (19/24) of the isolates, followed by the *tetA* gene [46.7% (14/30)]. The frequencies of the other non- β-lactam encoding genes include *sulI* and *sulII* [35.5% (11/31)], *tetB* [23.3% (7/30)], *aadA* [12.9% (4/31)], *tetC* [10% (3/30)], and *tetD* [3.3% (1/30)]. The different denominators indicate the number of the phenotypes for each antibiotic class for which the respective genes were assayed.

**Table 3:** Antibiotic resistance genotypes patterns in Enterobacteriaceae isolated from hospital wastewaters.

S/N	Antibiotic resistance genotypes	No. of ESBL genes	No. of pAmpC genes	No. of non-β-lactam encoding genes	No. of the observed pattern
	*Enterobacter* spp.				
1	*sulI*-*sulII*-c*atII*	0	0	3	1
2	*bla*_SHV_-*SulI*-*tetA*-*tetB*-*tetC*-*tetD*-*aadA*	1	0	6	1
3	*bla*_TEM_-*catII*	1	0	1	1
4	*tetA*	0	0	1	2
	*Klebsiella* spp.				
1	*bla*_TEM_-*bla*_OXA-1-like_-*bla*_CTX-M-1_-*sulI*-*sulII*-c*atII*-*tetA*-*tetB*	3	0	5	1
2	*bla*_TEM_-*bla*_OXA-1-like_-*bla*_CTX-M-1_-*bla*_CTX-M-2_-*bla*_CTX-M-9_-*bla*_FOX_-*bla*_CIT_-*sulI*-*sulII*-*catII*-*tetA*-*tetB*	5	2	5	1
3	*bla*_IMP_—*catII*	1	0	1	1
4	*bla*_CTX-M-1_-*catII*-*tetA*-*tetB*	1	0	3	1
5	*tetA*	0	0	1	1
6	*catII*-*tetA*	0	0	2	1
7	*bla*_TEM_-*catII*-*tetA*	1	0	2	1
8	*bla*_CTX-M-1_- *sulII*-*catII*	1	0	2	1
9	*catII*	0	0	1	1
10	*bla*_TEM_-*bla*_OXA-48-like_-*bla*_SHV_-*bla*_PER_-*bla*_VIM_- *bla*_EBC_-*bla*_CIT_- *sulI*-*tetB*-*tetC*	5	2	3	1
11	*bla*_TEM_-*bla*_OXA-48-like_-*bla*_OXA-1-like_-*bla*_PER_-*bla*_VIM_- *bla*_EBC_-*bla*_CIT_- *sulI*-*tetB*-*tetC*	5	2	3	1
12	*bla*_TEM_-*bla*_OXA-1-like_-*bla*_SHV_-*bla*_IMP_-*bla*_CTX-M-1_-*bla*_CTX-M-2_- *bla*_FOX_-*sulI*-*sulI*I-*tetA*-*tetB*	6	1	4	1
13	*catII*	0	0	1	2
14	*bla*_FOX_-*catII*-*tetA*	0	1	2	1
15	*bla*_TEM_	1	0	0	1
16	*bla*_CTX-M-9_-*sulII*-*catII*-*aadA*	1	0	3	1
17	*bla*_TEM_-*bla*_SHV_-*bla*_CTX-M-1_-*bla*_EBC_-*bla*_CIT_-*sulI*-*sulII*-*aadA*	3	2	3	1
18	*bla*_CTX-M-1_-*sulII*-*catII*-*tetA*	1	0	3	1
	*Citrobacter freundii*				
1	*bla*_CTX-M-1_-*tetA*	1	0	1	1
	*E*. *coli*				
1	*bla*_TEM_-*bla*_OXA-1-like_-*bla*_CTX-M-1_-*bla*_CTX-M-2_-*bla*_CIT_- *sulI*-*sulII*-*catII*-*tetB*-*tetC*-*tetD*-*aadA*	4	1	7	1
2	*bla*_OXA-1-like_-*bla*_CTX-M_-*sulI*-*sulII*-*catII*-*aadA*	2	0	4	1
3	*bla*_CTX-M-2_-*sulI*-*sulII*-*catII*-*tetA*	1	0	4	1

Fifteen β-lactamase resistance determinants were detected out of the 19 resistance determinants investigated. The four resistance determinants not detected include *bla*_GES_, *bla*_KPC_, *bla*_ACC,_ and *bla*_MOX_. Out of the 12 non-β-lactam resistance determinants assessed, four genes undetected were the *catI*, *strA*, *tetK*, and *tetM*. These genes belong to phenicols, aminoglycosides, and tetracyclines, antimicrobial classes, respectively. Of the 31 MDR bacterial isolates, 28 carried at least one of the resistance genes assessed. There were only three isolates that did not have any of the genetic determinants assayed. These were one *K*. *pneumoniae* (isolate No. 19) and two of the *C*. *freundii* (isolate No. 27 and 28) seen in Fig 2. Diverse genotypic patterns were detected, as shown in Table 3. Worthy of note is that majority of the genotypes occurred uniquely. With the exception noted only in *E*. *kobei* and *E*. *amnigenus*, both harbouring only the *tetA* gene (Isolate No. 4 and 5) and two of the *K*. *pneumoniae* isolates (Isolate No. 18 & 22) carrying only the *catII* gene as seen in Fig 2 and Table 3.

## Discussion

Hospitals are a crucial focus of AMR emergence and dissemination because there is a large-scale antibiotic use in the hospital environment. Many of these antibiotics are excreted non-metabolized from the body, thereby contributing to increased antibiotic residues and ARB in hospital wastes and municipal WWTPs [29,30], thereby making hospital effluents a potential route for the transfer of ARB and ARGs into the natural environment.

The confirmed isolates from the hospital effluents in this study revealed the dominance of the combination of *K*. *oxytoca* (11/31; 36%) and *K*. *pneumoniae* (9/31; 29%), with the former being more predominant among the eight species investigated. This result is akin to what was obtained in wastewater from Poland hospitals, where *K*. *pneumoniae* and *K*. *oxytoca* were among the most commonly recovered species among the Enterobacteriaceae [6]. Although *K*. *pneumoniae* is generally regarded as the most widespread hospital-acquired species of the genus [31], the high detection of MDR *K*. *oxytoca* recovered in this study is noteworthy and relevant. One *K*. *oxytoca* isolated was phenotypically resistant against all the 18 tested antibiotics. *K*. *oxytoca* causes infections and persists in the environment through biofilms [32,33]. In this study, all the *Klebsiella* spp. (n = 20) recovered exhibited phenotypic resistance to the third-generation antibiotics, cefotaxime and cefuroxime. These isolates also displayed a 100% resistance against ampicillin, ciprofloxacin, trimethoprim-sulfamethoxazole, and nalidixic acid. The analysis of the results obtained from a Brazillian hospital effluent equally revealed that several cephalosporin-resistant *Klebsiella* spp. (n = 8) displayed resistance against the cefotaxime and ceftazidime [34]. We report other high resistance frequencies noted. These include gentamicin (95%), tetracycline (95%), doxycycline (90%), chloramphenicol (90%), norfloxacin (90%), amoxicillin/clavulanic acid (85%). This trend is similar to a report from Kwazulu-Natal Province in South Africa, where most *Klebsiella* spp. (n = 72) isolated from hospital effluents were resistant against fluoroquinolone, aminoglycosides, and β-lactams [1]. In this study, the lowest resistance frequencies were observed in the antibiotic class carbapenems with a rate of 20% and 25% against imipenem and meropenem, respectively. This low resistance profile among the carbapenems is not surprising, as they are regarded as the last recourse of antibiotics for serious infections triggered by Enterobacteriaceae. Other similar studies have also reported resistance frequencies between 7% and 15% against carbapenems among their *Klebsiella* spp. [1,34].

Furthermore, *E*. *coli* and *C*. *freundii* were recovered in this study with a frequency of 10%, *E*. *asburiae* with 6%, while *E*. *hormaechei*, *E*. *kobei*, and *E*. *amnigenus* with 3%. In a related study on the hospital sewage in Taiwan, *Citrobacter* spp. (10.3%), *Klebsiella* spp. (11.3%), *Enterobacter* spp. (19.8%) and *E*. *coli* (32.9%) were the predominant taxonomic category within the 435 bacterial species recovered [35]. In another study, the genera isolated were Enterobacter (4%), Klebsiella (8%), Citrobacter (25.3%), and Escherichia (49.3%) out of 150 Enterobacteriaceae strains [6]. These results give insight into the consortium of Enterobacteriaceae members that are frequently isolated from hospital wastewater systems. Although the order of prevalence of species isolated is in contrast with our study, there is a relative comparativeness to the species identified in these effluents from different countries.

In this study, the *Enterobacter* spp. is the following prevalent genera among the four genera investigated among the Enterobacteriaceae. The two well-known species, *E*. *aerogenes* and *E*. *cloacae*, which are more commonly implicated in opportunistic nosocomial infections [36], were, however, not detected in this study. *Enterobacter* spp. have been described to serve as a reservoir of ARGs. There have been reports of these species acquiring various mobile genetic elements that confer certain fitness advantages, ensuring their ability to adapt to several environments. All the *Enterobacter* spp. isolated in this study were phenotypically resistant against β-lactams, cephems, tetracyclines, and aminoglycosides (Fig 2). These obtained phenotypes further corroborate the reports that *Enterobacter* spp. are intrinsically resistant against ampicillin, amoxicillin-clavulanic acid, and cefoxitin [36,37]. Surprisingly, only a few of the ARGs assayed were detected in this study. Among the β-lactamases, only *bla*_TEM_ and *bla*_SHV_ were detected, as seen in Table 3. This somewhat low occurrence of ARGs observed is similar to a previous report [38].

*E*. *coli* is one of the extensively studied and frequently isolated bacterial species in clinical and environmental studies. In this study, only three bacterial strains of *E*. *coli* were recovered. All the isolates displayed resistance against most of the 11 classes of antibiotics studied, including the carbapenems. However, they were all susceptible to chloramphenicol, resulting from this antibiotic’s low usage due to various potentially dangerous reported side effects. We report the detection of a variety of ARGs. The *E*. *coli* recovered harboured most of the non-β-lactam encoding genes (7). *Citrobacter* spp. have also been reported in the last decade to be resistant to the most generally used antibiotics, including ampicillin, cefotaxime, aminoglycoside, and tetracyclines [39]. In this study, only *C*. *freundii* was recovered (n = 3). They were all phenotypically resistant against β-lactams, cephems, tetracyclines, sulphonamides, aminoglycosides. These species were all susceptible to imipenem and meropenem. Therefore, these carbapenems may still be efficient against infections arising from *C*. *freundii*. Only one of these isolates carried two of the ARGs assayed (*bla*_CTX-M-1_ and *tetA*), which contrasts the results of the various β-lactamases reported in previous clinical and environmental studies [10,38–40].

The results in Tables 1 and 2 show various resistance phenotypes belonging to the critically essential antibiotics’ classes. The resistance against fluoroquinolones, aminoglycosides, β-lactams, and cephalosporins is quite problematic. Mainly, all the isolates in this study displayed resistance against these critically essential antibiotics’ classes described by the WHO [41]. The results indicate that this study’s bacterial isolates demonstrated a very high degree of resistance against most examined antibiotics. The MARI value obtained ranged from 0.5 to 1.0, which significantly exceeds the maximum MARI value benchmarked at 0.2 [27]. The high MARI values suggest that the bacterial isolates were retrieved from an environment with a very high selective pressure of antibiotics resistance. The result of this present study corroborates with other findings from hospital effluents, where 48.4% (150/310) of the Enterobacteriaceae isolated displayed phenotypic resistance to the antimicrobial agents investigated [6]. Most of the phenotypes observed were unique. MDR bacteria have been known to utilize various resistance mechanisms to evade the effects of antimicrobial agents used against them. Some of these mechanisms include the enzymatic modification of target sites, decreased permeability, possession of efflux pumps, and enzymes that inactivate antimicrobial agents [17,42].

In the Enterobacteriaceae group, the principal means of antimicrobial resistance is the ability to produce β-lactamases. These enzymes comprise the ESBLs and the pAmpC, which they harness in breaking the chemical structural backbone, the β-lactam ring, of the largest and commonly used antibiotic classes [17]. From the results of this study indicated in Table 3, these isolates’ ability to possess the ESBL/pAmpC enzymes is quite concerning. This scenario implies the undermining of existing antibiotics’ efficacy and could also hinder the development of new ones. In this research, the *bla*_CTX-M_ (51.6%) and the *bla*_TEM_ (40%) ESBL genes were the two most common types identified. This result is synonymous with the reported dominance of *bla*_CTX-M_ in the various hospital effluents [6,43], clinical isolates [44,45], and isolates from human waste [46]. The *bla*_CTX-M_ group has been reported as the most predominant enzymes among the β-lactamases, followed by the *bla*_TEM_ [40,47]. The CTX-M variants are one of the most widespread in South Africa [10,19]. As per this study, the co-occurrence of the ESBLs and pAmpCs were mostly detected in *Klebsiella* spp., with a similar co-carriage detected in one *E*. *coli* strain. Earlier researches indicated that ESBL-mediating plasmids might harbour over one resistance determinant [44]. Notably, two of the isolates carried 12 of the resistance genes. Fig 2 and Table 3 show that one *K*. *oxytoca* (isolate No. 7) had five ESBL genes, two pAmpC genes, and five non-β-lactam resistance determinants.

Similarly, one *E*. *coli* harboured four ESBL genes, one pAmpC gene, and seven non-β-lactam resistance determinants. Most of the genotypes occurred uniquely, indicating the possibility of the isolates acquiring unique and differing antimicrobial resistance genes. In this present study, carbapenemase resistance determinants, including *bla*_OXA-48-like,_ *bla*_VIM_, and *bla*_IMP_ that are commonly found in clinical settings, were also detected. The variants *bla*_KPC_, *bla*_GES,_ and *bla*_*O*XA-48-like_ have been reported in South African hospitals [11], although this study did not detect *bla*_KPC_ and *bla*_GES_. The detection of these carbapenemase ESBLs in hospital wastewater is quite worrisome primarily because of their possible eventual presence in the environment. This is because these hospital wastewaters are being discharged without any prior treatment into municipal WWTPs, which releases their effluents into receiving water bodies. Our previous study reported the presence of some of these critical enzymes in freshwater sources [10].

A high rate of detection of the non-β-lactam encoding genes was also noted. All the genes in this category detected occurred in combination with the β-lactamase resistance determinants except one *Enterobacter* spp., which harboured non-β-lactam encoding genes only (*sulI*—*sulII*–*catII*). The most prevalent non-β-lactam resistance gene detected was the *catII* gene, followed by the *tetA* gene. As seen in Table 3, the high prevalence of ARGs among the β-lactams and the non-β-lactams indicates that the β-lactam, phenicol, tetracycline, sulfonamide antibiotic classes are more frequently used antimicrobial agents in the hospital facilities where we carried out this research. Other researches have demonstrated that these antibiotics classes are commonly administered as first-line antimicrobials in medical applications. After consumption, the non-metabolized portion of these antibiotics will be excreted into the waste systems. The Enterobacteriaceae members present in the wastewater are then exposed to sublethal concentrations of multiple antimicrobial agents, consequently leading to their acquisition of various ARGs. The discharge of wastewater laden with potentially pathogenic MDR Enterobacteriaceae harbouring various ARGs, as seen in this study, constitutes an essential concern for people’s health. Hospital effluents are discharged without any prior treatment to municipal WWTPs, which in turn discharge their effluent to surrounding surface water. Surface water is usually abstracted for irrigating agricultural produce in developing countries, thereby potentially transferring these ARB and ARGs into the food chain.

## Conclusion

This study revealed the existence of multidrug-resistant Enterobacteriaceae’ members in hospital wastewater and the presence of a repertoire of various β-lactamases and other vital non-β-lactam resistance determinants which can be easily transferred to other organisms in multiple niches. The widespread of antibiotic resistant bacteria and their genes in hospital wastewater could, in turn, aggravate their occurrence within the environment as they are being discharged into the WWTPs. The WWTPs, even when functioning at optimal capacity, may be unable to curb the spread of antibiotic resistant bacteria and their genes into the environment, presenting a severe public health risk. Further studies need to be carried out to evaluate the incidence of antibiotic resistant bacteria and their genes in the municipal WWTPs to which hospital wastewater are discharged for treatment and access the efficiency of the same for the removal of these antimicrobial resistance determinants before discharge into the environment.

## Supporting information

S1 Table Primers used for screening extended-spectrum β-lactamase, plasmid AmpC determinants [48] as well as non-β-lactam resistance determinants [49] in Multidrug-resistant Enterobacteriaceae recovered from hospital wastewater. (PDF)

S2 Table MALDI-TOF identification of presumptive isolates obtained from hospital wastewater. (PDF)
