# Supplementary material for: Distribution and molecular characterization of ESBL, pAmpC β-lactamases, and non-β-lactam encoding genes in Enterobacteriaceae isolated from hospital wastewater in Eastern Cape Province, South Africa
Source: PLoS One. 2021 Jul 21;16(7):e0254753. doi: 10.1371/journal.pone.0254753 (PMC8294522; doi:10.1371/journal.pone.0254753)
Supplement: S1 Table — (PDF) [file pone.0254753.s001.pdf]

S1 Table. Primers used for screening extended-spectrum β-lactamase, plasmid AmpC determinants [48] **as well as non-β-lactam resistance determinants** [49] **in Multidrug-resistant Enterobacteriaceae recovered from hospital wastewater.**

Target genes	Primer sequences (5′ –3′)	Thermocycling conditions	Expected amplicon size (bp)
bla$_{\text{TEM}}$	F: CATTTCCGTGTCGCCCTTATTC	94 °C for 10 mins	800
	R: CGTTCATCCATAGTTGCCTGAC	30 cycles of	
bla$_{\text{SHV}}$	F: AGCCGCTTGAGCAAATTAAAC	94 °C for 40s	713
	R: ATCCCGCAGATAAATCACCAC	58 °C for 40s	
bla$_{\text{OXA-1-like}}$	F: GGCACCAGATTCAACTTTCAAG	72 °C for 1 min	564
	R: GACCCCAAGTTTCCTGTAAGTG	72 °C for 7 mins	
bla$_{\text{CTX-M Group 1}}$	F: TTAGGAARTGTGCCGCTGYA	94 °C for 10 mins	688
	R: CGATATCGTTGGTGGTRCCAT	30 cycles of	
bla$_{\text{CTX-M Group 2}}$	F:CGTTAACGGCACGATGAC	94 °C for 40s	404
	R: CGATATCGTTGGTGGTRCCAT	60 °C for 40s	
bla$_{\text{CTX-M Group 9}}$	F: TCAAGCCTGCCGATCTGGT	72 °C for 1 min	561
	R: TGATTCTCGCCGCTGAAG	72 °C for 7 mins	
bla$_{\text{PER}}$	F: GCTCCGATAATGAAAGCGT		520
	R: TTCGGCTTGACTCGGCTGA		
bla$_{\text{VEB}}$	F: CATTTCCCGATGCAAAGCGT		648
	R: CGAAGTTTCTTTGGACTCTG		
bla$_{\text{FOX}}$	F: CTACAGTGCGGGTGGTTT	94 °C for 10 mins	162
	R: CTATTTGCGGCCAGGTGA	30 cycles of	
bla$_{\text{DHA}}$	F:TGATGGCACAGCAGGATATTC	94 °C for 40s	997
	R: GCTTTGACTCTTTCGGTATTCG	59 °C for 40s	
bla$_{\text{CIT}}$	F: CGAAGAGGCAATGACCAGAC	72 °C for 1 min	538
	R: ACGGACAGGGTTAGGATAGY	72 °C for 7 mins	
bla$_{\text{EBC}}$	F: CGGTAAAGCCGATGTTGCG		683
	R: AGCCTAACCCCTGATACA		
bla$_{\text{IMP}}$	F: TTGACACTCCATTTACDG	94 °C for 10 mins	139
	R: GATYGAGAATTAAGCCACYCT	30X (94 °C for 40s; 55 °C for 40s; 72 °C for 1 min)	

bla$_{VIM}$	F: GATGGTGTTTGGTCGCATA<br>R: CGAATGCGCAGCACCAG	72 °C for 7 mins	390
bla$_{OXA-48-like}$	F: GCTTGATCGCCCTCGATT<br>R: GATTTGCTCCGTGGCCGAAA	94 °C for 10 mins<br>30X (94 °C for 40s; 57 °C for 40s; 72 °C for 1 min)<br>72 °C for 7 mins	281
aadA	F:GTGGATGGCGGCCTGAAGCC<br>R:AATGCCCAGTCGGCAGCG	94 °C for 4 mins<br>30X (94 °C for 45s; 50 °C for 45s; 72 °C for 45s)<br>72 °C for 7 mins	525
tetA	F:GCTACATCCTGCTTGCCTTC<br>R:CATAGATCGCCGTGAAGAGG	94 °C for 5 mins<br>35 cycles of	201
tetB	F: TTGGTTAGGGGCAAGTTTTG<br>R:GTAATGGGCCAATAACACCG	94 °C for 1 min<br>55 °C for 1 min	359
tetC	F:CTTGAGAGCCTTCAACCCAG<br>R:ATGGTCGTCATCTACCTGCC	72 °C for 1.5 mins<br>72 °C for 5 mins	418
tetD	F:AAACCATTACGGCATTCTGC<br>R:GACCGGATACACCATCCATC		300
sul I	F:TTCGGCATTCTGAATCTCAC<br>R:ATGATCTAACCCTCGGTCTC		822
sul II	F:CGGCATCGTCAACATAACC<br>R:GTGTGCGGATGAAGTCAG	94 °C for 5 mins<br>30 cycles of	625
catII	F:ACACTTTGCCCTTTATCGTC<br>R:TGAAAGCCATCACATACTGC	94 °C for 30s; 50 °C for 30s; 72 °C for 1.5 mins<br>72 °C for 5 mins	543

[a]Annealing position within the corresponding open reading frame (from the base A of start codon ATG).

[b]Y=T or C; R=A or G; S=G or C; D=A or G or T
