# Supplementary material for: Distribution and molecular characterization of ESBL, pAmpC β-lactamases, and non-β-lactam encoding genes in Enterobacteriaceae isolated from hospital wastewater in Eastern Cape Province, South Africa
Source: PLoS One. 2021 Jul 21;16(7):e0254753. doi: 10.1371/journal.pone.0254753 (PMC8294522; doi:10.1371/journal.pone.0254753)
Supplement: S2 Table — (PDF) [file pone.0254753.s002.pdf]

S2 Table. MALDI-TOF identification of presumptive isolates obtained from hospital wastewater.

Family	Genus	Species	Number identified
Moraxellaceae	*Acinetobacter*	*johnsonii*	**4**
Enterobacteriaceae	*Citrobacter*	*freundii*	3
	Enterobacter	*amnigenus*	1
	Enterobacter	*asburiae*	2
	Enterobacter	*hormaechei*	1
	Enterobacter	*kobei*	1
	Escherichia	*coli*	3
	Klebsiella	*oxytoca*	11
	Klebsiella	*pneumoniae*	9
	Serratia	*marcescens*	3
	Raoultella	*ornithinolytica*	1
			**35**
	Not reliable identification		**5**
	**GRAND TOTAL**		**44**
